# Advances in omics data for eosinophilic esophagitis: moving towards multi-omics analyses

**DOI:** 10.1007/s00535-024-02151-6

**Published:** 2024-09-19

**Authors:** Kazuhiro Matsuyama, Shingo Yamada, Hironori Sato, Justin Zhan, Tetsuo Shoda

**Affiliations:** 1 Division of Allergy and Immunology, Cincinnati Children’s Hospital Medical Center, Department of Pediatrics, University of Cincinnati College of Medicine, 3333 Burnet Avenue, MLC 7028, Cincinnati, OH 45229 USA; 2 Department of Computer Science, University of Cincinnati, Cincinnati, USA; 3 Department of Pediatrics, Graduate School of Medicine, Chiba University, Chiba, Japan

**Keywords:** Eosinophilic esophagitis, Machine learning, Multi-omics

## Abstract

Eosinophilic esophagitis (EoE) is a chronic, allergic inflammatory disease of the esophagus characterized by eosinophil accumulation and has a growing global prevalence. EoE significantly impairs quality of life and poses a substantial burden on healthcare resources. Currently, only two FDA-approved medications exist for EoE, highlighting the need for broader research into its management and prevention. Recent advancements in omics technologies, such as genomics, epigenetics, transcriptomics, proteomics, and others, offer new insights into the genetic and immunologic mechanisms underlying EoE. Genomic studies have identified genetic loci and mutations associated with EoE, revealing predispositions that vary by ancestry and indicating EoE’s complex genetic basis. Epigenetic studies have uncovered changes in DNA methylation and chromatin structure that affect gene expression, influencing EoE pathology. Transcriptomic analyses have revealed a distinct gene expression profile in EoE, dominated by genes involved in activated type 2 immunity and epithelial barrier function. Proteomic approaches have furthered the understanding of EoE mechanisms, identifying potential new biomarkers and therapeutic targets. However, challenges in integrating diverse omics data persist, largely due to their complexity and the need for advanced computational methods. Machine learning is emerging as a valuable tool for analyzing extensive and intricate datasets, potentially revealing new aspects of EoE pathogenesis. The integration of multi-omics data through sophisticated computational approaches promises significant advancements in our understanding of EoE, improving diagnostics, and enhancing treatment effectiveness. This review synthesizes current omics research and explores future directions for comprehensively understanding the disease mechanisms in EoE.

## Introduction

Eosinophilic esophagitis (EoE) is an allergic condition characterized by inflammation of the esophageal mucosa and an accumulation of eosinophils [1, 2]. Though historically considered rare, EoE prevalence is increasing worldwide [3, 4]. Patients with EoE endure considerable clinical burden—the lowest quality of life compared with a series of other chronic pediatric diseases [5], and EoE is a substantial challenge to healthcare systems in both resource utilization and associated costs [6]. EoE is a persistent disease from childhood into adulthood, progressing to fibrostenotic complications—esophageal scarring and narrowing—that can be relatively refractory to therapy [7, 8]. Currently, only two medications have FDA approval for treating EoE (i.e., dupilumab [9] and budesonide oral suspension [BOS] [10]), emphasizing the urgent need for expanding EoE prevention and management research [11]. Understanding the mechanism of EoE manifestation is crucial, including delving into the molecular mechanisms at play and identifying disease markers that could predict diagnosis and outcomes.

**Fig. 1:**
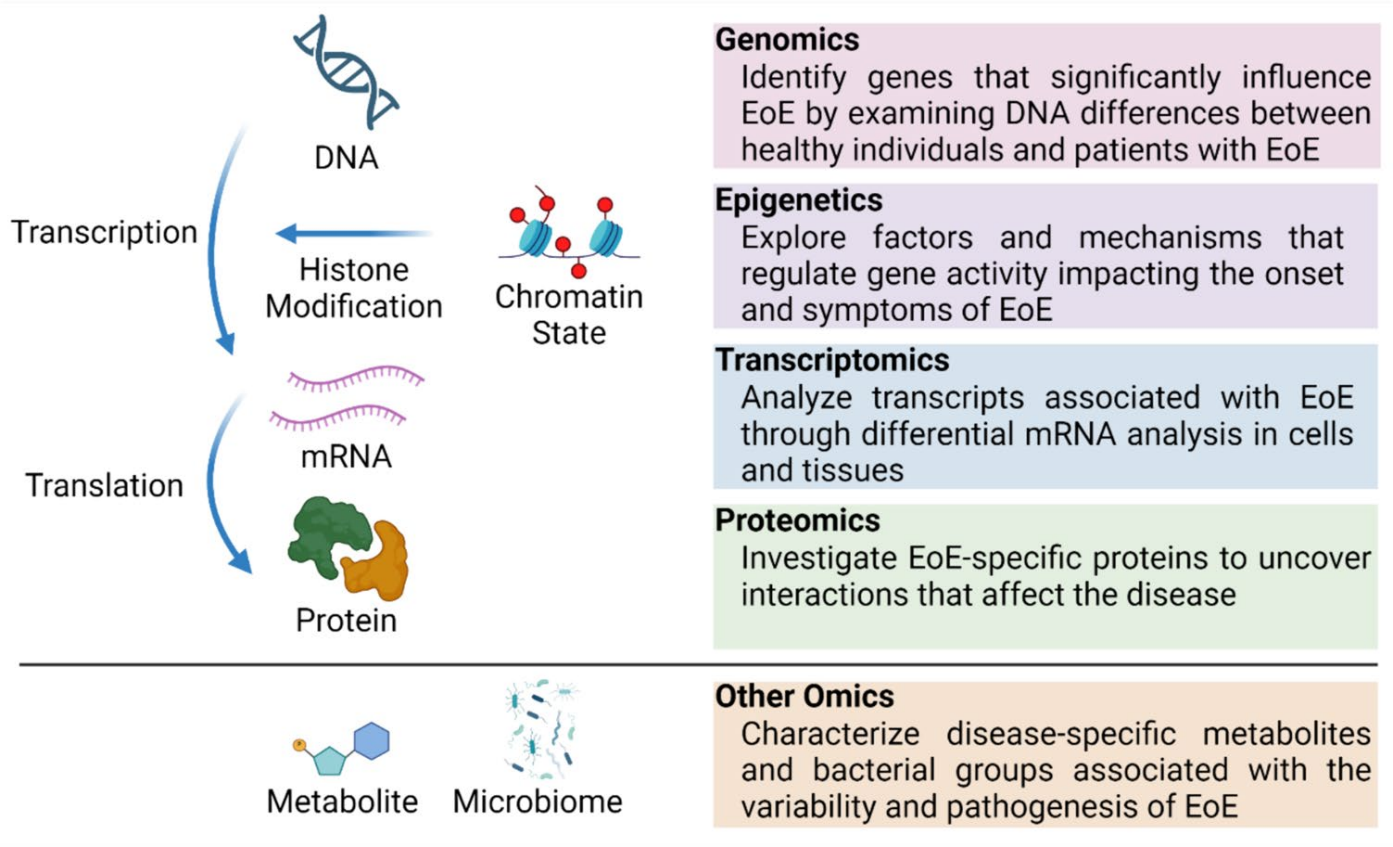
Overview of simplified omics data. *EoE* eosinophilic esophagitis. Created with Biorender.com

**Fig. 2:**
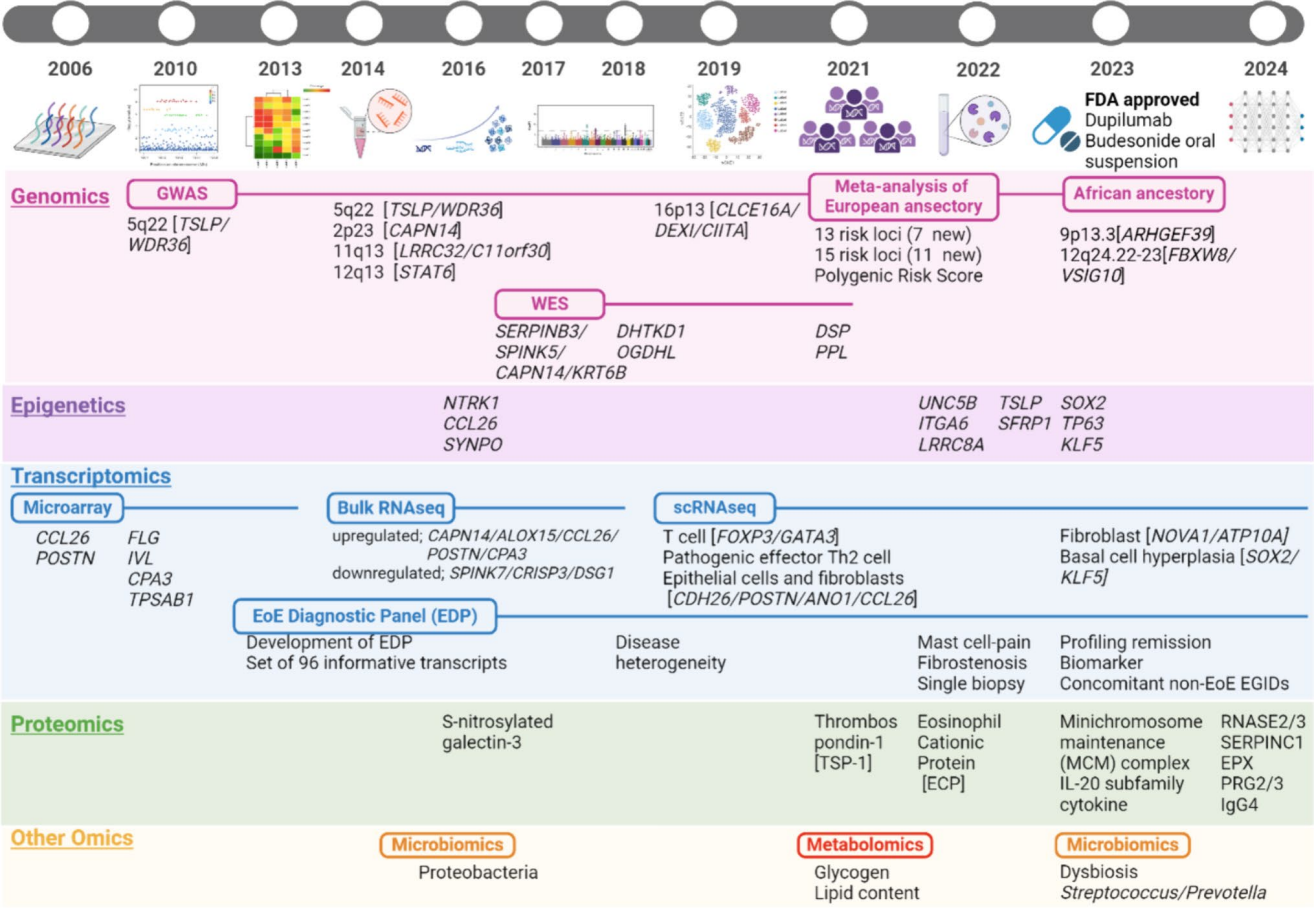
Schematic summary for the timing of relevant events in the genetic and molecular progress in EoE. A chronological list of genes and proteins found by Genomics, Epigenetics, Transcriptomics, Proteomics, and other omics related to EoE. *EoE* eosinophilic esophagitis, *GWAS* genome-wide association studies, *WES* whole exome sequencing, *RNAseq* RNA sequencing, *scRNAseq* single-cell RNA sequencing. Created with Biorender.com

**Fig. 3:**
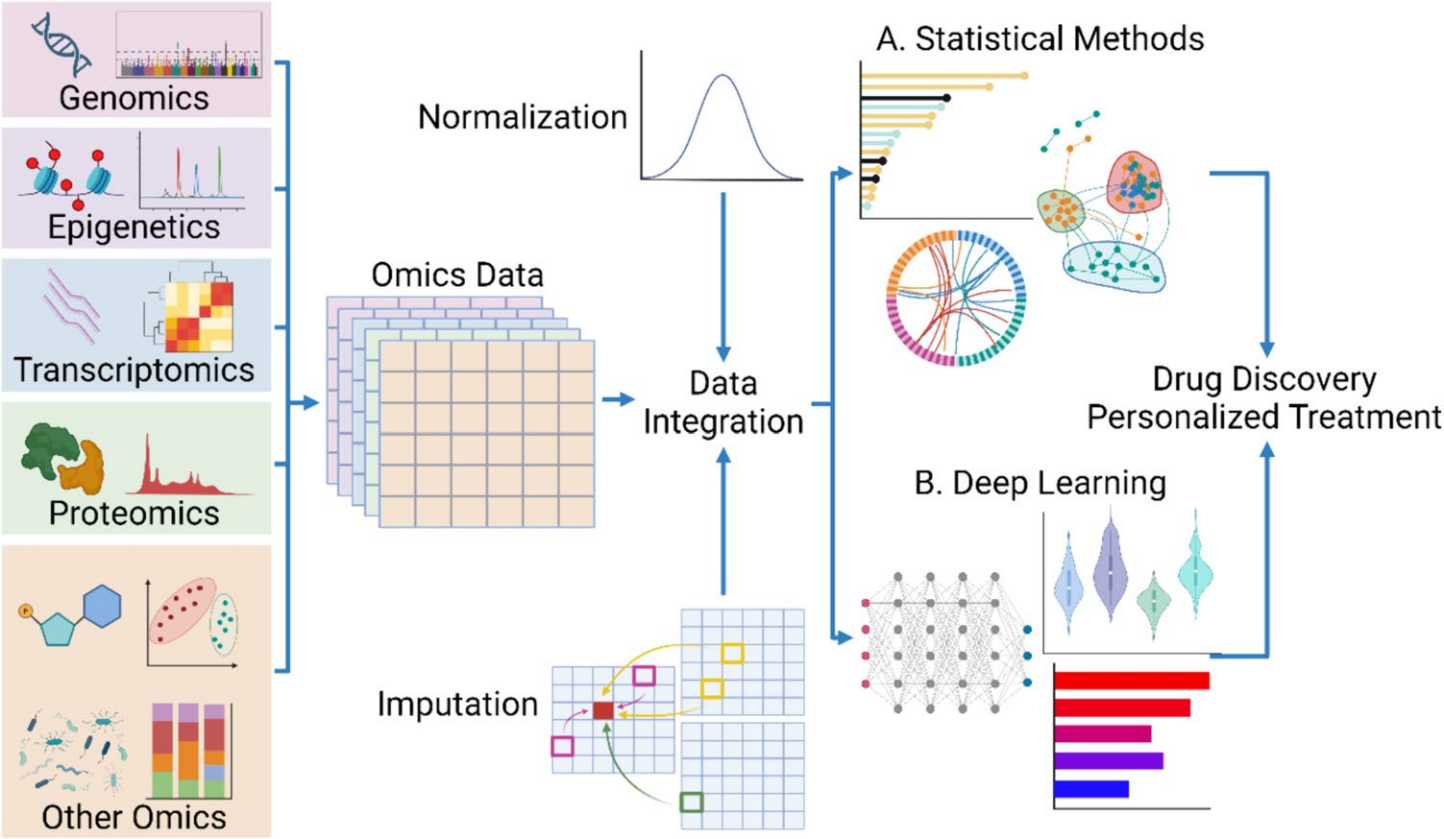
Integrative schema of multi-omics studies of EoE. Data-integration framework in EoE characterized by the combination of heterogeneous information, including multi-omic datasets. Data of each omics data reintegrated and analyzed by two methods. **A** Statistical Methods: unsupervised learning is performed by determining explanatory variables from the analysis of each omics data and incorporating them in the model. **B** Deep Learning: disease-specific factors are found by extracting features from each omics data. Data integration can also be done after feature extraction through a deep learning model. *EoE* eosinophilic esophagitis. Created with Biorender.com

The recent advancements in the field of “omics”—including genomics, epigenetics, transcriptomics, proteomics, and other comprehensive approaches to study large sets of biological data—offer promising new opportunities for understanding diseases at a molecular level. For EoE, omics technologies, leveraging high-throughput sequencing, have begun to elucidate the intricate interplay of genetic and immunologic factors that contribute to the disease [12, 13] (Figs. 1, 2). These technologies enable researchers to examine the complex genetic and immunologic interactions that underlie EoE, providing a more comprehensive understanding of the disease than was previously possible. Despite the valuable insights from omics studies, effectively integrating individual omics into multi-omics is still in its early stages. Challenges include the scarcity of comprehensive datasets and the need for advanced computational tools to analyze and interpret these complex data layers. Machine learning technologies [14] are one promising approach to overcome these hurdles, as they can analyze large datasets quickly and with high accuracy, potentially revealing new aspects of EoE pathogenesis (Fig. 3).

In this review, we consolidate current knowledge from various omics studies related to EoE and discuss the advantages and challenges of integrating individual omics into multi-omics approaches using machine learning technologies to explore future directions in understanding the mechanisms underlying EoE.

## EoE genomics

Genomics, the science of studying genomes, began with DNA sequencing and has rapidly expanded to include exploring gene and protein expression profiles and their functional roles [15]. These advances have significantly improved our understanding of various diseases, including EoE. Recent studies highlight the complex genetic influences on EoE, revealing higher prevalence rates among males, Caucasians, and younger individuals [12, 16]. Notably, EoE frequently occurs within families, particularly among siblings, and follows a non-Mendelian inheritance pattern, indicating a complex genetic foundation [13]. This section specifically discusses the insights into EoE genomics provided by genome-wide association studies (GWAS) and whole exome sequencing (WES) (Fig. 2, Genomics).

### EoE GWAS

GWAS typically report blocks of correlated single-nucleotide polymorphisms (SNPs), known as genomic risk loci, that are statistically associated with the trait of interest [17]. A major strength of GWAS is their ability to systematically and unbiasedly search for novel disease-associated variants.

Loci associated with EoE pathogenesis have been identified by GWAS and are presented in chronological order. A pioneering EoE GWAS of 181 children with EoE and 170 normal controls, all of European ancestry, found an important genetic association with chromosome 5q22 [18]. This region encompasses *TSLP*/*WDR36*. *TSLP* encodes thymic stromal lymphopoietin, a cytokine promoting allergic inflammation. *TSLP* was found to be highly expressed in EoE, underscoring the pivotal role of *TSLP* in EoE pathogenesis and potential therapeutic targets. Subsequently, a larger GWAS was performed from > 1.5 million genetic markers in EoE [19], greatly expanding the number of EoE cases and controls compared to the previous study. The genetic association of 5q22 (*TSLP/WDR36*) was confirmed, and a new association with 2p23 (*CAPN14*) was identified. *CAPN14* encodes calpain 14, a calcium-activated cysteine protease, and is highly tissue specific; it is expressed in esophageal epithelium with little expression in the epithelium of other organs. *CAPN14* is activated by IL-13 and contributes to maintaining and repairing epithelial tissue [19]. Notably, *CAPN14* was also confirmed by another GWAS [20]. In addition to *CAPN14*, 11q13 (*c11orf30/LRRC32*) and 12q13 (*STAT6*), previously reported as associated with atopy and autoimmune diseases, and 19q13 (*ANKRD27*), which regulates the transport of melanogenic enzymes to epidermal melanocytes, were newly identified [20]. The risk locus 16p13 (*CLEC16A/DEXI/CIITA*), which is expressed in both immune cells and esophageal epithelial cells, also was identified, further expanding the EoE genetic landscape [21].

Meta-analyses have broadened our understanding of GWAS studies, especially in overcoming the limitations posed by small sample sizes in rare diseases such as EoE. At present, four GWAS and two meta-analysis of GWAS identified 41 tag variants corresponding to nine genome-wide significant loci and 27 suggestive loci [18–23]. The first GWAS meta-analysis identified replicated association at 6 loci from 627 EoE and 365 controls having European ancestry: 2p23 (2 independent genetic effects), 5q22, 10p14, 11q13, and 16p13 [22]. Another seven loci were identified with suggestive significance at 1q31, 5q23, 6q15, 6q21, 8p21, 17q12, and 22q13, leading to identifying 13 protein-coding EoE risk gene candidates. To assess the genetic risk of individual loci, the Polygenic Risk Score (PRS) was created, and the genetic burden of GWAS-identified EoE risk loci were assessed. Those with the highest genetic burden had a 12-fold greater risk of developing EoE than those with the lowest genetic burden [22]. Another meta-analysis, including 1,930 affected European subjects and 13,634 ancestry-matched controls, identified 15 genome-wide significant EoE risk loci, 11 of which were novel; three loci, 5q31.1 (*RAD50*), 15q22.2 (*RORA*), and 15q23 (*SMAD3*), may have a pivotal role in EoE development [23]. Though these two meta-analyses were conducted on European ancestry, there are also reports with 137 Black and African-American cases of EoE and 1,465 healthy controls [24]. The proportion of African ancestry was found to be significantly lower in EoE than in controls, and three significant EoE-associated loci were identified (9p13.3 [*ARHGEF39*], 12q24.22–23 [*FBXW8/VSIG10*], 15q11.2), of which 12q24.22–23 and 9p13.3 were recapitulated in the case–control analysis and found to be associated with African ancestry.

### EoE WES

Although GWAS has identified multiple common EoE-associated SNPs, a challenge for GWAS, especially for rare conditions like EoE, is that rare variants (allele frequencies < 1%) are excluded due to power concerns [25]. In contrast, WES comprehensively targets all exonic regions of genes to uncover critical mutations, proving especially valuable for rare genetic diseases such as EoE [13]. Though GWAS primarily identifies mutations in non-coding regions, WES has revealed several rare protein-coding mutations, enhancing our understanding of EoE genetic underpinnings.

In particular, previous studies have identified protein-coding mutations within EoE. For instance, a study involving 33 unrelated patients with EoE revealed 39 rare mutations across 18 esophagus-specific genes, including *SERPINB3, SPINK5, CAPN14*, and *KRT6B*, with notable mutations also found in *GABRP* in four individuals [26]. Functional analyses of these mutations indicated a strong involvement in biological processes like epidermal cell differentiation and serine-type endopeptidase inhibitor activity, suggesting a critical role for these esophagus-specific genes in EoE pathogenesis. Another WES study utilized family-based trio analysis for 37 unrelated families, including 63 patients with EoE and 60 unaffected family members [27]. Dehydrogenase E1 and transketolase domain-containing 1 (*DHTKD1*) and oxoglutarate dehydrogenase L (*OGDHL*) were identified as EoE-involved genes, highlighting their potential pathogenic role in EoE mitochondrial dysfunction. Furthermore, a recent WES of an extended multi-generational family identified rare heterozygous missense mutations in genes encoding the desmosome-associated proteins DSP and PPL [28]. These mutations, found in 21% of polygenic families, primarily affected the esophageal squamous epithelium and were implicated in altering barrier integrity, cellular motility, and Rho GTPase activity. These findings enhance our understanding of the tissue-specific mechanisms that may underlie EoE allergic reactions.

Overall, WES has illuminated the significant impact of rare genetic variants in EoE, underscoring EoE’s multifactorial nature and the substantial effect sizes that these rare variants may have. This insight not only advances our understanding of EoE pathogenesis, but also aids in the potential development of targeted therapies.

### Limitations and future potential

Despite the reduced sequencing costs and advances in bioinformatics enhancing the identification of EoE-associated genetic variants, genomics still faces notable challenges. Although there have been some reports of gene–environment interactions [29], these remain insufficient for a comprehensive understanding of complex diseases like EoE. Additionally, the PRS shows great promise in clinical applications [22]; however, it has yet to reach practical utility in clinical settings. As GWAS sample sizes increase and PRS accuracy improves, these tools are expected to significantly influence research and personalized medicine. Moreover, the predominance of GWAS conducted in populations of European descent limits the broader applicability of the findings across diverse ethnic groups. Enhancing GWAS cohort diversity and leveraging advances in bioinformatics and machine learning will be key to enabling more personalized EoE management and treatment, ensuring that findings are applicable globally.

## EoE epigenetics

Epigenetics is an emerging field in genomic research, primarily examining how alterations in chromatin structure can either enhance or suppress DNA transcription [30]. The aim is to identify methylation sites, promoters, or enhancers that affect DNA transcription and to elucidate disease mechanisms. DNA-Methylation [31], ChIP-seq [32], and ATAC-seq [33] have advanced our understanding of genome-wide epigenetic marks and transcription factor binding sites [34]. Methylation analysis examines the expression status of specific genes by identifying methylation sites in DNA. ATAC-seq employs a hyperactive Tn5 transposase to cleave open chromatin for high-throughput sequencing, requiring fewer cells than traditional methods [35]. ChIP-seq uses antibodies to identify enriched DNA loci and analyze histone modifications, categorizing genomic regions into distinct chromatin states and examining motif sequences to infer transcription factor bindings [36], either by discovering new motifs or scanning existing ones [37]. ChIP-seq also analyzes functional enrichment to associate nearby genes with potential biological pathways. We summarize these approaches for EoE as follows (Fig. 2, Epigenetics).

Predicting responses of EoE to topical corticosteroid treatment identified molecular markers for specific CpG sites [38]. Logistic regression analysis on biopsy samples and DNA methylation data from 88 patients pinpointed significant CpG sites associated with treatment outcomes. After adjusting for covariates, results showed that specific CpG sites (cg26152017 in *UNC5B*, cg01044293 in *ITGA6*, and cg13962589 in *LRRC8A*) were significantly associated.

Genome-wide DNA methylation of 20 children aged 4–16 years with and without EoE identified the 25 methylated CpG loci as potential biomarkers to distinguish between patients with EoE and healthy controls [39]. Furthermore, principal component analysis discovered differences in methylation profiles that correlated with diagnosis, eosinophil count, and age. These loci not only facilitated EoE diagnosis, but also maintained distinct profiles from healthy controls, even after eosinophil counts decreased. This finding highlights their potential utility in monitoring disease progression and diagnosis.

Quantitative analysis, such as Chip-PCR [40], found association of EoE with T helper type 2 (Th2) allergic reactions [41]. In EoE, IL-13 significantly upregulated 15 genes, with 10 of these genes displaying active epigenetic marks on their promoters, including *NTRK1* and *CCL26*. The *SYNPO* gene, coding for an actin-related protein, was notably influenced by IL-13, showing increased isoform expression. This isoform upregulation impacts cell motility, barrier integrity, and differentiation in esophageal epithelial cells, underscoring a crucial role of *SYNPO* in EoE pathology driven by IL-13–mediated transcriptional and epigenetic changes.

ATAC-seq analysis of EoE biopsy tissue identified 798 loci with altered chromatin structures, indicating significant epigenetic modifications [42]. Th2 cells, which differentiate from T cells and secrete type 2 cytokines, are essential for sustaining allergic responses [43]. TSLP was an early cytokine expressed in the activated epithelium after mucosal barrier disruption [44]. TSLP induced Th2 cell differentiation and robust intracellular cytokine production and proliferation [42]. This TSLP action contributes to EoE pathology, with the increased percentage of CD4 + T cells responding to TSLP in the blood serving as an EoE diagnostic tool.

A recent study [45] evaluated 41 EoE gene risk variants from independent EoE disease risk loci obtained in preceding studies [18–21, 25, 46]. From these risk variants, 30 allele enhancer variants for 3 cell types were identified. Using expression quantitative trait loci (eQTLs) [47] of cell-cultured tissue sections with the EoE allele enhancer variants revealed 219 genes presumed to be regulated by these enhancers [45]. These analyses yielded 6 EoE risk loci as important alleles. In addition, rs2289277, an eQTL associated with genotype-dependent *TSLP* expression, was an allelic enhancer variant in IL-13–stimulated TE-7 cells. This finding suggests a mechanistic basis for the previously reported elevated *TSLP* expression.

Accumulating epigenetic analyses have uncovered a relationship between EoE and STAT motifs [48]. STAT3 strongly correlated with IL-13-induced esophageal epithelial proliferation and expression of EoE proliferation genes. Tissue-cultured EoE biopsies and IL-13-stimulated esophageal epithelial cells expressed 82 differential genes, with about half exhibiting STAT motifs. The relationship between STAT1, STAT3, STAT4, STAT5a, and STAT6 and transcription factors was analyzed using ChIP-X Enrichment Analysis [49]; 32 of the genes targeted STAT3, establishing STAT3 involvement. Nine genes associated with STAT6, suggesting that STAT6 was not relevant to esophageal epithelial growth [48]. However, STAT6 signaling by IL-13 regulates the inflammatory response of the esophagus. Gene Ontology (GO) analysis [50] identified *SFRP1* as an important regulator of IL-13-induced and STAT3-dependent esophageal proliferation and basal zone hyperplasia (BZH) in EoE [48].

Previous discovery of transcription factors stored in the public databases have highlighted BZH involvement in EoE [51]. A single-cell transcriptome of EoE biopsy tissue and enrichment analyses utilizing either the ChEA3 2022 ChIPSeq database [52] or the enrichR-provided libraries [53–55] captured dysfunctional, non-proliferating basal clusters of esophageal epithelial cells. Examining those cells’ differentiation process found *SOX2*, *TP63*, and *KLF5* as differentially expressed genes in the constructed esophageal epithelial differentiation cluster for enrichment and upstream regulators of stem cell self-renewal. As *KLF5* was recently identified as a *SOX2* binding partner [56], the *SOX2* and *KLF5* interaction was further investigated [51]. Pseudo-temporal analysis indicated that *SOX2* and *KLF5* expression increased over time and distinct characteristics of EoE epithelial cells, particularly in superficial esophageal layers.

### Limitations and future potential

With the growing interest in disease-relevant epigenetic changes, many innovative analytical methods have been developed. When using multi-omics data, appropriate handling of data acquired by different methods is key [14]. Because of the different acquisition methods, these data cannot be treated equivalently, and interpretations may need to be treated independently. For example, integrating separate CpG methylation data and microRNA data requires that both data be acquired from the same sample. Another way to address this challenge is to use known biological pathways or publicly available datasets to obtain estimates. Although this approach can complement missing data, its accuracy needs to be validated.

## EoE transcriptomics

The transcriptome encompasses all RNA in a cell or group of cells, reflecting dynamic changes across developmental stages or in response to specific conditions [57]. Since the 1990s, the technologies for transcriptomics have progressed from microarray technology to bulk RNA sequencing (RNAseq) and more recently to single-cell RNA sequencing (scRNAseq), each offering more detailed and unbiased views of gene expression under various conditions. Indeed, these advances have revealed a unique EoE transcriptional signature, aiding in the understanding of EoE’s complex gene regulation and functions. This section discusses how these transcriptomic insights have advanced our understanding of EoE pathogenesis (Fig. 2, Transcriptomics).

### Identifying distinct transcript signatures

A significant advancement in understanding EoE occurred when gene expression analysis of esophageal biopsies identified a unique transcript signature that differentiates patients with EoE from healthy controls and those with chronic esophagitis. Since the first microarray-based transcriptome analysis in 2006 [46], several studies utilizing microarray and RNAseq have been conducted across different cohorts [58–63]. These studies have consistently identified a certain set of differentially expressed genes that constitute the EoE transcriptome. Remarkably, this transcriptome maintains a high degree of consistency across variations in patient sex, age, and atopic history, and it shows a strong correlation with esophageal eosinophil levels [46].

Among the EoE transcriptome, the most highly upregulated gene is the eosinophil chemoattractant eotaxin-3 (*CCL26*) [64]. As a crucial member of the CC chemokine family, *CCL26* interacts with its receptor, *CCR3*, to activate G protein signaling pathways, significantly enhancing eosinophil chemotaxis and activation. Unlike other eotaxins, *CCL26* is uniquely upregulated in EoE, establishing a clear association with eosinophil levels in esophageal biopsies [46]. *CCL26* increase can be found in eosinophilic gastritis (EoG) and duodenitis but not eosinophilic colitis [2, 65–67].

Beyond eosinophil-related genes, the EoE transcriptome also exhibits differential expression of various immune cell-specific genes, including those associated with mast cells [58]. Among them, *CPA3* and *TPSAB1* are highly expressed in the EoE esophagus, underscoring the significant involvement of mast cells in the inflammatory response. The esophageal transcriptome specific to mast cells only partially overlaps with that defined by eosinophil levels, suggesting that mast cells and eosinophils contribute independently to EoE pathology. This distinction is supported by recent clinical trials in which EoE symptoms persisted despite eosinophil-depleting therapies [68, 69], indicating the need for further research to explore the distinct roles of these immune cells in EoE.

A significant portion of the transcriptional changes occur within the esophageal epithelium, affecting inflammatory cell recruitment, tissue remodeling, and hyperproliferation [70]. The non-keratinized, stratified squamous epithelium of the esophagus displays significant histopathologic changes in EoE, such as dilated intercellular spaces and an expanded BZH. These alterations are significantly driven by IL-13, which modulates gene expression and can replicate the EoE transcriptome in ex vivo studies [59]. The epidermal differentiation complex on chromosome 1q21, crucial for epithelial differentiation and barrier function, also features prominently in the EoE transcriptome. This includes genes like filaggrin (*FLG*) and involucrin (*IVL*), which exhibit unique expression patterns in the esophageal epithelium [59]. Additionally, the transcriptome is enriched in genes related to proteases and the IL-1 family, underscoring innate immunity involvement [26]. Notably, decreased expression of the serine protease inhibitor *SPINK7* exacerbates proteolytic activity and inflammation, providing key insights into EoE pathogenesis [71].

Furthermore, certain transcriptomic changes persist despite EoE remission, particularly involving genes associated with fibrosis and tissue remodeling including genes like periostin (*POSTN*) [72, 73]. Periostin is a component of the extracellular matrix that interacts with IL-13, mainly produced by Th2 cells and mast cells, and TGF-β, mainly produced by eosinophils and mast cells [74]. These interactions result in a dramatic upregulation of *POSTN* expression in esophageal fibroblasts, which contributes to the persistent fibrosis observed in the EoE lamina propria [75]. The sustained high levels of periostin promote eosinophil adhesion, collagen synthesis, and fibrotic responses, reinforcing a feedback loop that exacerbates tissue remodeling [73]. These dynamics highlight the complexity of managing fibrosis in EoE and underscore the need for targeted therapeutic strategies addressing these specific molecular mechanisms.

Regarding the application of transcriptomics, the development of the Eosinophilic Esophagitis Diagnostic Panel (EDP) significantly advanced the clinical use of transcriptome analysis [76]. This 96-gene qPCR array not only excels at distinguishing patients with EoE controls, but also facilitates RNA analysis from formalin-fixed or paraffin-embedded tissues, thereby reducing the need for repeated biopsies. The EDP enabled predicting inflammation and detecting relapses from a single biopsy [77], understanding disease heterogeneity [78], identifying connections between pain and mast cells [79], deciphering the mechanisms of fibrostenosis [80], characterizing a similar molecular signature between EoE and esophageal involvement [81], and profiling remission markers [72]. The EDP has substantially improved the diagnosis and understanding of EoE pathophysiology, and future efforts will focus on exploring genes not currently included in the EDP to enhance its accuracy and clinical utility.

Collectively, transcriptome analysis technologies have established EoE as a distinct disease with unique molecular profiles and provided valuable insights into key molecules that contribute to various changes observed in EoE.

### scRNAseq

scRNAseq has provided profound insights into EoE pathogenesis, revealing complex interactions among various cell types at the cellular level, which bulk RNAseq cannot delineate due to its analysis of mixed cell populations [82].

The first scRNAseq study in EoE involved 17 patients with active EoE and 6 normal controls, focusing on specific T cells [83]. This study highlighted a significant enrichment of CD4 + regulatory T cells (FOXP3 +) and Th2 cells (GATA3 +) within tissue-residing CD3 + T cells, localizing type 2 cytokine production to these effector populations and suggesting a role for *FFAR3* in amplifying local Th2 responses in EoE. Further scRNAseq analyses have shown an increase in pathogenic effector Th2 (peTH2) cells, enriched in the NF-κB signaling pathway, that associates with esophageal eosinophil levels in patients with active disease [84].

Analysis of esophageal epithelial cells revealed six major cell lineages, with specific markers identifying subpopulations such as epithelial cells, lymphocytes, myeloid cells, mast cells, endothelial cells, and fibroblasts [85]. Notably, esophageal epithelial cells and fibroblasts demonstrated upregulation of genes critical in EoE pathogenesis, such as *CDH26*, *POSTN*, *ANO1*, and *CCL26*, whereas downregulated genes were primarily expressed in the epithelial components.

A recent study on fibroblasts identified cell type–specific expressions of EoE risk genes like *NOVA1* in the esophageal fibroblasts and *ATP10A* in the PRDM16 + dendritic cells enriched during active disease [86]. BZH, characterized by abnormal increases in *SOX2* and *KLF5* expression, was observed in EoE but not in reflux esophagitis, indicating that reflux does not simply cause BZH [51].

Overall, advances in scRNAseq are unveiling detailed and accurate transcriptomic data at the single-cell level, providing deeper insights into the immune mechanisms, epithelial barrier functions, and remodeling processes in EoE. This technology is paving the way for a better understanding of the disease’s cellular and molecular mechanisms.

### Limitations and future potential

Traditional transcriptome analysis has provided valuable insights into EoE, identifying key molecular signatures and differentially expressed genes. However, this method often loses critical spatial information once RNA is extracted, obscuring how cells interact within their environments. Spatial transcriptomics offers a solution by mapping gene expression directly within tissue sections, capturing subtle cellular interactions and variations [87]. Integrating this method into EoE research could revolutionize our understanding by identifying precise biomarkers for diagnosis, predicting therapeutic responses, and monitoring disease progression. Combining spatial transcriptomics with scRNAseq could unveil detailed molecular and cellular interactions, potentially leading to targeted treatment strategies tailored to individual EoE profiles.

## EoE proteomics

Proteomics involves the large-scale study of proteins to understand their structures and functions [88]. Bottom-up proteomics based on mass spectrometry has emerged as a critical research technique for comprehensive protein analysis. This method has seen rapid advances in both device technology and data analysis, making it increasingly accessible for a wide range of diseases (e.g., cancer, immune diseases) [89–92]. To uncover novel biomarkers for disease severity, differentiate between diseases, and provide deeper mechanistic insights, proteomics is now being applied to EoE; however, studies are still relatively scarce. This section explores the types of proteomics and techniques recently used in EoE research, highlighting significant findings in pathologic analysis and biomarker discovery (Fig. 2, Proteomics).

Most proteomics studies utilize mass spectrometry–based techniques, such as high-performance liquid chromatography-mass spectrometry (HPLC–MS), tandem mass spectrometry (MS/MS), and matrix-assisted laser desorption/ionization time-of-flight MS/MS (MALDI–TOF MS/MS) to analyze proteome composition [93, 94]. These methods involve purifying and digesting proteins from biological samples, identifying them by analyzing spectral data from ionized peptides with a mass spectrometer, and matching these data to known databases [95].

EoE proteomic analyses have emerged to advance our understanding of disease mechanisms, histologic changes such as BZH, epithelial barrier, and fibrosis. For instance, the minichromosome maintenance (MCM) complex, associated with proliferative epithelial cells, was found to be significantly expressed in inflamed esophageal tissues, with inhibition experiments (ciprofloxacin) highlighting MCM as a potential therapeutic target [96]. Another proteomic research study, by combining transcriptomic and functional analyses, uncovered elevated levels of IL-20 subfamily cytokines in active EoE, suggesting these cytokines as novel therapeutic targets due to their role in downregulating barrier-protective genes like filaggrin [97]. Furthermore, using proteomics analyses of diseased and normal esophageal fibroblasts cultured on autologous or opposing derivative extracellular matrixes, thrombospondin-1 was discovered and validated as a pathogenic mediator of EoE fibrosis [98].

Comparative proteomic analyses have attempted to identify potential biomarkers for EoE. Research has revealed heightened expression of S-nitrosylated galectin-3 in the esophageal mucosa of patients with eosinophilia, suggesting its potential as a biomarker [99]. Additionally, liquid chromatography-tandem mass spectrometry (LC–MS/MS) comparisons of esophageal biopsies from pediatric EoE, gastroesophageal reflux disease (GERD), and healthy controls identified eosinophil cationic protein (ECP) as significantly upregulated in EoE [100]. Furthermore, a study detected 363 differentially accumulated proteins in patients with EoE compared to healthy subjects, including eosinophil-associated proteins (e.g., RNASE2, RNASE3, SERPINC1, EPX, and PRG3) that correlated with eosinophil counts and disease severity, proposing new, minimally invasive biomarkers [101]. Another study focused on the deposition of IgG4 and food proteins in the esophageal mucosa of patients with EoE, identifying specific eosinophil-derived proteins (e.g., PRG2, PRG3, EPX, and RNASE3) and calpain-14 in IgG4-enriched regions using the AutoSTOMP technique [102]. This research also confirmed the IgG4 binding to various food allergens, advancing our understanding of EoE’s immunologic responses and informing potential targeted treatment strategies. Taken together, these proteomic findings are instrumental in advancing EoE diagnosis and treatment.

### Limitations and future potential

Proteomics serves as a crucial tool for stratifying patients, identifying therapeutic targets, and discovering biomarkers. However, it faces several limitations that affect its utility. One major challenge is the variability in protein expression due to the severity of the disease and individual patient factors, especially noticeable in studies with small sample sizes, complicating data standardization. Additionally, the dynamic range of protein quantification in mass spectrometry-based proteomics is limited, which can restrict the detection of low-abundance proteins. Looking to the future, advanced proteomics techniques offer detailed protein information that may be undetectable at the mRNA level, bridging gaps left by transcriptome analysis. Recent technological advancements now permit the detection of protein numbers comparable to those identified in transcriptomic studies [103]. Furthermore, innovative methods like multiplex antibody assays, which utilize microliters of liquid samples to target specific proteins without mass spectrometry, are expanding the scope of detectable proteins [104]. These developments necessitate careful methods and sample size selection based on specific research goals. Moreover, the potential for proteomics to facilitate less invasive diagnostic approaches, such as liquid biopsies, is particularly promising in pediatrics where traditional endoscopic assessments for conditions like EoE are more invasive [89]. Liquid biopsy could revolutionize the way pathologies are studied and biomarkers are discovered, offering a minimally invasive option to collect vital diagnostic information. This approach holds great promise for enhancing phenotypic and pathologic analyses across various diseases.

## EoE other omics

### Metabolomics and microbiomics

The recent increasing incidence of EoE suggests that environmental factors may be influencing their development [29]. Metabolomics, which directly measure metabolites produced during cellular activities, can track pathologic changes and aid in managing diseases [105]. This approach is useful in food elimination therapy, where identifying causative foods is challenging; measuring metabolites can help assess treatment response and reduce patient burden. Microbiomics reveal how alterations due to modern lifestyles impact disease [106]. Factors such as drug use, breastfeeding status, and environmental exposures contribute to microbial imbalances that are closely linked to inflammation and disease progression. This section covers these two omics approaches separately (Fig. 2, Other Omics).

### Metabolomics

Metabolomics analysis is categorized into non-targeted and targeted methods. Non-targeted metabolomics aims to analyze a broad spectrum of metabolites from biological samples, utilizing techniques such as nuclear magnetic resonance and mass spectrometry. LC–MS/MS is widely used for its capability to detect a diverse range of metabolites, making it ideal for comprehensive metabolic profiling [107–110]. Targeted metabolomics focuses on specific biochemical pathways, employing methods such as spectroscopy and flame ionization to provide detailed characterization of specific metabolites. For example, Raman spectroscopy [111] is a nondestructive analysis of the chemical structure and interactions of materials. This approach offers deep insights into specific biochemical pathways, enhancing our understanding of metabolic functions.

Raman spectroscopy analysis has revealed metabolite-specific spectra associated with EoE in children [111]. The profile of the biochemical composition of esophageal samples from 24 children with and without EoE pinpointed spectral markers specific for EoE. Notably, Raman peaks related to glycogen content were lower in children with EoE than those without EoE. Additionally, the glycogen content correlated inversely to lipid content and to the severity of histopathology assessed by EoE HSS. These metabolites might serve as spectral markers indicative of EoE activity and the degree of pathology [112]. The study investigators proposed that this inverse correlation is primarily driven by the degree of eosinophilic inflammation, potentially linked to peri-epithelial cells and BZH [111].

### Microbiomics

Microbiomics comprises metagenomics, metatranscriptomics, and metabolite-based metabolomics [113]. Metagenomics involves sequencing the DNA of cells from biopsies and swab specimens and mapping the genes of the microbial community; it assesses microbial pathways and abundance, but activity and contribution in disease is difficult to assess [114]. Metatranscriptomics reveals activity in the environment by identifying expressed transcripts in the microbiome. Metabolomics evaluates changes in microbial metabolites, such as lipids, carbohydrates, and amino acids, and analyzes biochemical changes associated with disease phenotypes; however, it is not suitable for identifying microbial community types [105]. These complementary methods contribute to validating the efficacy of treatments, identifying environmental differences, and elucidating effects on pathways. This section mainly focuses on metagenomic analysis.

The disease activity in EoE influenced the composition of the esophageal microbiota [115]. When comparing microbiota of esophageal biopsies from 33 pediatric subjects with EoE and 35 non-EoE pediatric controls, a characteristic esophageal microbiome in EoE that is influenced by EoE disease activity was shown. Distinctive microbiota such as enrichment of Proteobacteria, including Neisseria and Corynebacterium, in the esophagus were reported in active EoE compared to non-EoE controls. Also, comparing the esophageal inflammation characteristic of EoE with inflammation due to other diseases revealed differences in bacterial communities in the esophageal mucosa [116]. The esophageal string tests of individuals who were healthy and those who had treated EoE disease showed the association of bacterial communities in the esophageal mucosa with treatments. Although an increase in bacterial abundance was observed in active EoE, the difference in bacterial community composition between treated and untreated EoE was limited, with active EoE having Haemophilus significantly increased in the esophagus. Unlike active EoE, GERD esophageal disease activity was not associated with increased bacterial load in the EoE.

Different treatment choices for EoE, including steroid therapy, proton pump inhibitors (PPIs), and dietary modifications, have been shown to affect esophageal microbial composition [117, 118]. Diversity analysis and clustering have revealed that patients with EoE in remission from these treatments exhibit distinct microbial patterns. For example, the steroid treatment group displayed a unique microbial composition. However, a comparison of the esophageal microbiomes of 49 adults with and without EoE found no significant correlations between the microbiome and endoscopic findings, such as exudates, ring changes, edema, grooves, stricture, or esophageal dilatation. These results imply that the esophageal microbiota, at the time of diagnosis in adults with EoE, may not influence the disease’s pathophysiology.

Examining a large cohort revealed certain prominent taxa in both the esophagus and stomach, reflecting environmental biases [119]. "Streptococcus" and "Prevotella" were identified as dominant in the EoE and EoG samples, respectively. In addition, an increase in taxa with Gram-negative cell wall structure was observed in the EoE samples. This expansion of taxa with Gram-negative cell wall structures may influence the inflammatory process and suggests that this may be an important feature in EoE pathogenesis.

A mouse model of EoE has provided insights into how microbiota colonization influences esophageal morphology and gene expression, highlighting pathways particularly related to epithelial barrier function [120]. An abnormal microbiota, characterized by the absence of lactobacilli, associated with key changes involving genes such as *POSTN, KLK5*, and *HIF1*, indicating a disrupted esophageal microenvironment. After fecal microbiota transplantation (FMT), Streptococcaceae were not detected in the esophagus of any recipient mice. The absence is consistent with natural transient variations in colony formation. Moreover, it demonstrated how esophageal microbiota in germ-free mice recovered post FMT. These findings are helpful for advancing our understanding of esophageal health and pathology.

### Limitations and future potential

The field of metabolomics faces the future challenge of conducting large-scale quantitative analyses in EoE, which currently lacks such studies. Additionally, integrating microbiome data with other omics data presents specific challenges, including biases introduced by variable environmental factors, which can skew results across different datasets. The gut microbiome—a diverse community of microorganisms—plays a pivotal role in immune system development and metabolic activity, influencing the onset of digestive and atopic diseases. Factors like drug use, breastfeeding status, and environmental exposure contribute to microbial imbalances, which are closely linked to inflammation and disease progression. Some studies have explored the impact of PPI exposure on the local microbiome and the interaction between toll-like receptors (TLRs), such as TLR4, and lipopolysaccharides [121–123]. However, it is unclear whether changes in the microbiome initiate inflammation or result from pathology. Further analysis with metabolites produced by microbiome and integrating other omics will deepen new discoveries.

## EoE treatment and omics

The results of the omics analysis in EoE contributed to the development of treatments and are used to validate their efficacy. PPIs and allergen elimination diet have been typical initial treatment. They have been evaluated for efficacy by omics analysis [124–126]. The elucidation of biological mechanisms through omics analysis is driving the development of new molecule-targeted drugs beyond these two therapies. For example, the anti-IL-5 antibodies mepolizumab, reslizumab [127, 128], the anti-IL-5Rα antibody benralizumab [69], the anti-IL-13 antibodies cendakimab [129] and dectrekumab [130], the anti-TSLP antibody tezepelumab [131], and the anti-SIGLEC-8 antibody lirentelimab [132] are also in clinical trials.

Since 2022, the FDA has approved two drugs for EoE: dupilumab in May 2022 [9, 133] and BOS in February 2024 [10] (Fig. 2, FDA approved). It is remarkable that dupilumab, a monoclonal antibody against IL-4Rα, improved dysphagia, change in peak eosinophil count, endoscopic severity, histologic severity, and esophageal distensibility compared to placebo and that the EoE transcriptome was reversed [133]. There is no omics evaluation for BOS, but it has been reported that daily administration of high-dose fluticasone propionate resulted in histologic remission in 65–77% of patients with EoE after 3 months and that the gene expression pattern was similar to that of patients without EoE [134]. In the future, omics analysis will further elucidate the molecular pathophysiology of EoE, which will in turn facilitate the development of new drugs. Integration of clinical manifestations, histologic responses, and omics data will be essential in the evaluation of new drugs.

## Integrating omics in EoE (multi-omics)

Large-scale quantitative analysis using next-generation sequencers has enhanced the integrated analysis of omics data. The integration has been achieved through statistical analysis, utilizing classifications such as genome-first, phenotype-first, and environment-first to identify statistically significant pathways [135]. In recent success of machine learning, multi-omics integration has evolved to include deep learning models, which offer broad interpretability. These models typically employ one of two approaches based on the sequence of omics data integration and training, addressing challenges posed by incomplete datasets and missing values. This advancement is crucial, helping to understand complex biological interactions. This section explores the multi-omics integration strategies employed in EoE studies, highlighting their potential to uncover novel insights into the disease.

### Multi-omics analysis

The development of the EoE TaMMA [136], derived from the Multi-Omics Factor Analysis (MOFA) framework [137] has elucidated a predominance of microbiota abnormalities in EoE pathogenesis. Through the analysis of biological processes using transcriptomics and meta-transcriptomics profiling, specific microbial signatures that distinguish EoE from GERD and controls were identified. Advanced deconvolution techniques, such as MuSiC [138] and CIBERSORTx [139], were utilized to further refine the datasets. Importantly, bacterial species distinctively abundant in EoE were identified, with 9 candidates emerging as specific markers for EoE in the esophagus. Additionally, the analysis enabled the development of 4 multilayered molecular signatures that effectively differentiate EoE patients from controls. These findings point to microbial dysbiosis as a key factor in EoE pathogenesis. This framework proves highly applicable for integrating various omics datasets, providing a starting point for further multi-omics studies in EoE.

Integrated analysis of transcriptomics and metabolomics has linked transcriptomic signatures to specific metabolites and immune components in French children with EoE [140]; utilizing liquid chromatography coupled with high-resolution statistical analysis via the DIABLO model [141] identified 4 key plasma immune components and 8 metabolites that significantly associated with EoEs. Furthermore, supervised partial least square-discriminant analysis (PLS-DA) demonstrated high accuracy in predicting EoE status, providing a comprehensive view of both cellular and soluble immune components in esophageal biopsies from affected children and controls. The study also emphasized the potential benefits of translating these biopsy results into viable serologic tests that could assess the presence and/or severity of EoE, marking a prominent advance in diagnostic methodologies.

### Limitations and future potential

Recent developments in data integration research have advanced data integration strategies that combine data from multiple omics analyses. For instance, a study examined esophageal dysbiosis by integrating omics data [136]. A multimodal learning model that integrated biopsy tissue images and omics data technique was important for elucidating EoE genetic mechanisms [142–146]. Future studies should incorporate new analysis methods, such as spatial omics analysis [147] and Cut&Run [148], while integrating them with the vast amount of existing omics data. These new techniques are poised to extract meaningful information and provide insight on processes involved in developing EoE.

Data integration of omics data and extracting significant information has been done in the past, but there are two challenges [149]. The first is the comprehensiveness of the dataset, and the second is having enough data points to approach the true probability distribution of the objective variable. Overfitting to achieve apparent high accuracy or underfitting due to insufficient data can lead to a misguided understanding of diseases with complex mechanisms. However, the nature of rare diseases makes it difficult to prepare large datasets. A framework for rare diseases with small data sizes is urgently needed to approach the true probability distribution. In addition, compensating for deficiencies by collecting and reanalyzing existing publicly available data and performing more accurate deconvolution will be required in the future. The analysis of omics data and its comparison with in vivo and in vitro observations will aid in the future understanding of diseases.

The long-term prognosis of current treatments is not clear. Among the approved therapies, Dupilumab can be administered to children over 1 year of age but requires weekly injection [9], which raises concerns about its use as a treatment for children. BOS has not been proven safe and effective in the treatment of EoE beyond 12 weeks [10]. Data on the long-term effects of continuous administration are lacking, and these continued doses may increase the economic burden on patients and healthcare systems.

To address these issues, further understanding EoE molecular mechanisms is urgently needed: applying machine learning to predict molecular mechanisms and analyzing patient characteristics will impact future research results. These approaches can accelerate research by highlighting notable candidates for existing experimental methods. These technological advances will help to elucidate further mechanisms of the disease and lead to the development of therapeutics that will offer a fundamental cure.

## Conclusion

In this review, we discussed advances in omics data for EoE. Elucidating the disease mechanism by each omics analysis has resulted in FDA approval of two therapeutic drugs. In the future, further development is expected in delineating disease mechanisms through each omics analysis and multi-omics analysis using tissue imaging data and other omics data.

## Abbreviations

ATAC-seq: Assay for transposase-accessible chromatin using sequencing
BOS: Budesonide oral suspension
BZH: Basal zone hyperplasia
ChIP-seq: Chromatin immunoprecipitation sequencing
CpG: Cytosine phosphate guanine
DIABLO: Data integration analysis for biomarker discovery using latent components
ECP: Eosinophil cationic protein
EDP: Eosinophilic esophagitis diagnostic panel
EoE: Eosinophilic esophagitis
EoG: Eosinophilic gastritis
eQTLs: Expression quantitative trait loci
FDA: United States of America Food and Drug Administration
FMT: Fecal microbiota transplantation
GERD: Gastroesophageal reflux disease
GO: Gene ontology
GWAS: Genome-wide association studies
HPLC–MS: High-performance liquid chromatography‒mass spectrometry
HSS: Histology Scoring System
LC–MS/MS: Liquid chromatography‒tandem mass spectrometry
MALDI–TOF MS/MS: Matrix-assisted laser desorption/ionization time-of-flight tandem mass spectrometry
MCM: Minichromosome maintenance
MOFA: Multi-omics factor analysis
MS/MS: Tandem mass spectrometry
MuSiC: Multi-subject single-cell deconvolution
peTH2: Pathogenic effector T helper type 2
PLS-DA: Supervised partial least square-discriminant analysis
PPIs: Proton pump inhibitors
PRS: Polygenic risk score
RNAseq: RNA sequencing
scRNAseq: Single-cell RNA sequencing
SNPs: Single-nucleotide polymorphisms
TaMMA: Transcriptome and metatranscriptome meta-analysis
Th2: T helper type 2
TLRs: Toll-like receptors
WES: Whole exome sequencing
